# Wind tunnel measurement dataset of 3D turbulent flow around a group of generic buildings with and without a high-rise building

**DOI:** 10.1016/j.dib.2021.107504

**Published:** 2021-10-23

**Authors:** Yoshihide Tominaga, Mohammadreza Shirzadi

**Affiliations:** a Wind and Fluid Engineering Research Center, Niigata Institute of Technology, 1719 Fujihashi, Kashiwazaki, Japan; b Chemical Engineering Department, Graduate School of Advanced Science and Engineering, Hiroshima University, 1-4-1, Kagamiyama, Higashi Hiroshima, Japan

**Keywords:** Wind tunnel experiment, Velocity magnitude, Turbulent statistics, Gust factor, Urban blocks, High-rise building, Wind pressure coefficient, Pedestrian wind environment

## Abstract

This paper presents a high tempo-spatial resolution dataset of the three-dimensional (3D) turbulent flow over a group of generic buildings with and without a high-rise building measured in an atmospheric boundary layer wind tunnel. This dataset is the basis of the study reported in the research article entitled “Wind tunnel measurement of three-dimensional turbulent flow structures around a building group: Impact of high-rise buildings on pedestrian wind environment” by Tominaga and Shirzadi (2021), which investigates the effect of a high-rise building on the pedestrian wind environment formed around the surrounding buildings and its interaction mechanism with the street flow at the pedestrian height. The instantaneous velocity vectors over a vertical central plane and a horizontal plane and the time-averaged surface pressure over the central building were measured for two cases consisting of a low-rise (Case 1H) and high-rise (Case 3H) buildings, which are in the center of a group of eight low-rise cubic buildings at a regular arrangement with an urban planar area density of 0.25. Data acquisition procedure and measurements details are explained in this paper. Time-averaged values of three velocity components and surface pressure coefficients, and turbulent statistics, i.e. turbulent kinetic energy and normal component of the Reynolds stresses are presented. Furthermore, the time-averaged two-dimensional (2D) velocity magnitude over the pedestrian height are presented for evaluating the gust factor. The presented database is useful for the validation of computational fluid dynamics (CFD) models and turbulent model developments for urban and building-related studies.

## Specifications Table

**Table:** 

Subject	Engineering, Environmental Engineering
Specific subject area	Wind engineering, Building Engineering, Pedestrian comfort, Urban ventilation
Type of data	Table Image Figure
How data were acquired	Split fiber probe (SFP) (Dantec Dynamics; 55R55) and a constant temperature anemometry (CTA) module (Dantec Dynamics; 90C10) for measurement of three components of the velocity vector SFP (Dantec Dynamics; 55R56) and a CTA module (Dantec Dynamics; 90C10) for measurement of 2D velocity magnitude Multipoint transducer (Kyowa Electronic Instruments; F94–2206) for static pressure measurements
Data format	Raw Analyzed
Parameters for data collection	Two cases consisting of a low-rise (Case 1H) and high-rise (Case 3H) buildings (H: building height), which are in the center of a group of eight low-rise cubic buildings at a regular arrangement with an urban planar area density of 0.25, are considered under a perpendicular wind direction. Reynolds number based on the H and the reference velocity $U_{H}$ is $Re=2.1\;\times 10^{4}$.
Description of data collection	The sampling rate and sampling frequency for the velocity measurements were 60 s and 1000 Hz, respectively. The sampling rate and sampling frequency for the pressure measurements were 60 s and 100 Hz, respectively. Pressure and velocity measurements were conducted separately but within similar controlled conditions.
Data source location	Institution: Niigata Institute of Technology City/Town/Region: Kashiwazaki, Niigata Country: Japan
Data accessibility	With the article
Related research article	Y. Tominaga and M. Shirzadi, “Wind tunnel measurement of three-dimensional turbulent flow structures around a building group: Impact of high-rise buildings on pedestrian wind environment,” Build. Environ., vol. 206, p. 108,389, 2021: https://doi.org/10.1016/j.buildenv.2021.108389.

## Value of the Data

• The dataset is intended to provide a high tempo-spatial resolution database for assessing the wind environment around a group of buildings in presence of a high-rise building to assess the complex interactions of the flow around the high-rise building and the street flow.
• The dataset can be used by researchers and CFD users in the field of wind environment and urban climate simulations for validation studies.
• The dataset can be used as a benchmark for accuracy assessment of different CFD settings, including grid resolution, turbulence models, numerical schemes, steady-state or transient analysis [2], [3], [4].
• The dataset can be utilized as a high-quality source for calibration of the closure coefficients of turbulence models [5], [6], [7].

## 1 Data Description

The dataset presented in this paper are described in the following:

**Wind tunnel profile of the approaching flow (supplementary file Table 1):**

The wind tunnel profiles of the time-averaged streamwise velocity, turbulent kinetic energy, and fluctuating velocity components in the streamwise, lateral, and vertical directions, which are measured at the center of the empty turntable are shown in this file.

**Turbulent statistics over the vertical plane for Case 1H (supplementary file Table 2):**

This table shows the normalized values of the turbulence statistics over the vertical plane for Case 1H.

**Turbulent statistics over the horizontal plane for Case 1H (supplementary file Table 3):**

This table shows the normalized values of the turbulence statistics over the horizontal plane for Case 1H.

**Turbulent statistics over the vertical plane for Case 3H (supplementary file Table 4):**

This table shows the normalized values of the turbulence statistics over the vertical plane for Case 3H.

**Turbulent statistics over the horizontal plane for Case 3H (supplementary file Table 5):**

This table shows the normalized values of the turbulence statistics over the horizontal plane for Case 3H.

**Wind pressure coefficient for Case 1H and Case 3H (supplementary file Table 6):**

This table shows the time-averaged pressure coefficients $\left(C_{p}\right)$ for Case 1H and Case 3H.

**Gust factor for Case 1H and Case 3H (supplementary file Table 7):**

This table presents the gust factor (GF) at the pedestrian level (horizontal plane) for Case 1H and Case 3H.

The data presented within this paper are completed with the coordinates of the measurement points over horizontal and vertical planes for the velocity measurements for Case 1H and Case 3H, which are shown in Fig. 3 (see Section 3 of this paper).

## 2 Experimental Design, Materials, and Methods

### 2.1 Building configuration

**Fig. 1:**
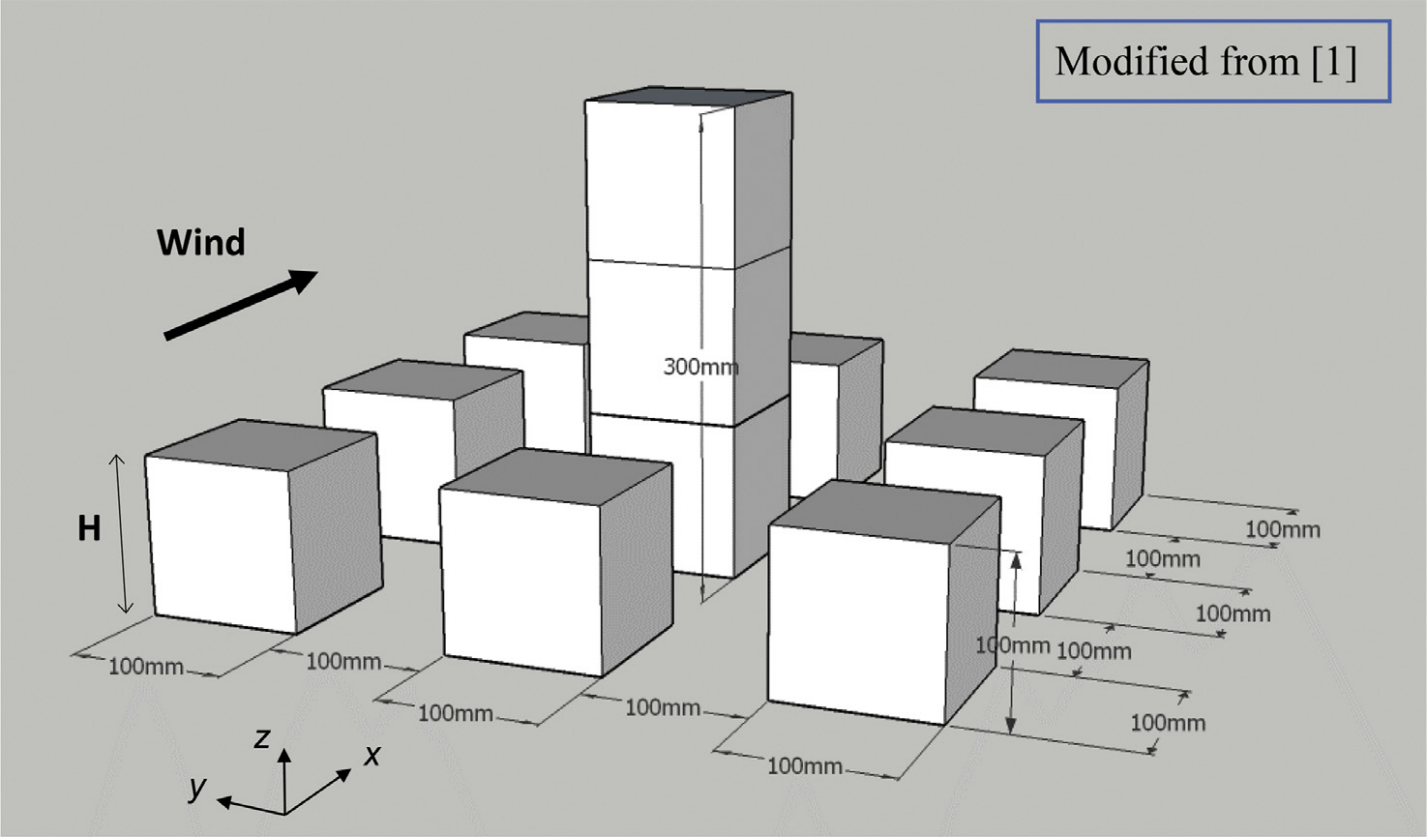
Building configuration.

The building configuration, as shown in Fig. 1, is a group of nine generic cubic buildings which are arranged in a regular form with a planar area ratio of $\lambda_{p}=0.25$. The planar area ratio is defined as:

(1) $$\lambda_{P}=\frac{BD}{\left(B+W\right)\left(D+W\right)}$$

where B and D are the breadth and depth of the building, respectively, and W is the distance between the buildings. The distance between all buildings in the lateral and streamwise directions is 0.1m.

Two different cases are considered: Case 1H and Case 3H. For Case 1H, the height of the central building is the same as the surrounding building's height, i.e. H=0.1m. For Case 3H, a high-rise building is considered as the central building with a height of 3H. These configurations are similar to test case C (simple city blocks) of the AIJ (Architectural Institute of Japan) benchmark [8,9]. Furthermore, this configuration was used in several CFD validation studies, e.g. [10], [11], [12].

### 2.2 Experimental settings

**Fig. 2:**
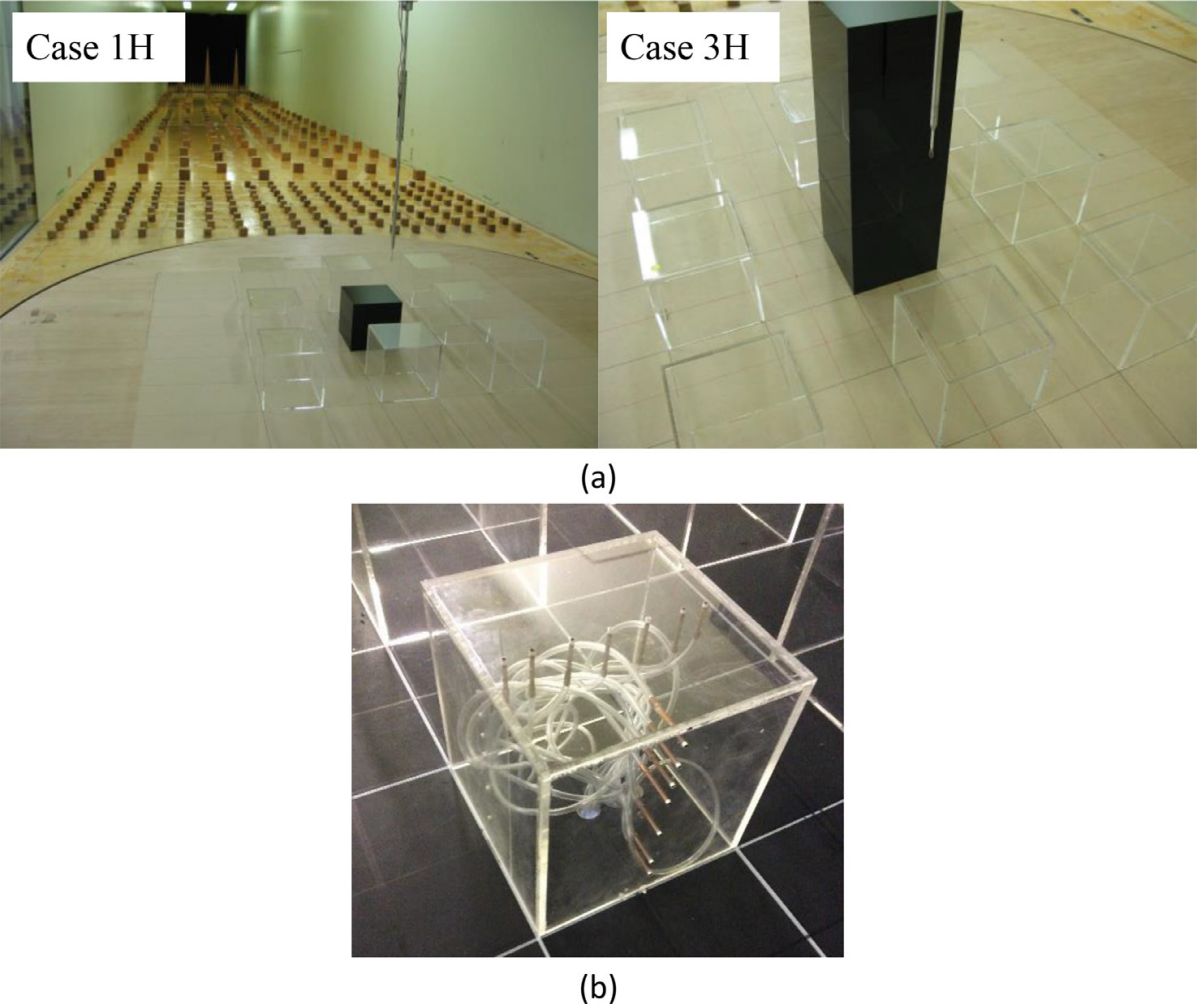
Measurement set-up for (a) velocity (modified from Tominaga and Shirzadi [1]) and (b) pressure measurements.

Experiments were performed in the atmospheric boundary layer wind tunnel at the Niigata Institute of Technology [13,14]. The test section length is 13m and the cross-section dimension is 1.8×1.8m. The experimental set-up for the velocity and surface pressure measurements is shown in Fig. 2. The building models were made of 3 mm-thick acrylic plates.

The reference velocity measured at the center of the empty turntable at H=0.1m is $U_{H}=3.1\frac{m}{s}$. The building Reynolds number, which is determined by H and $U_{H}$, is $2.1\;\times \;10^{4}$. The aerodynamic roughness length $z_{o}$, deduced from the line fitted to the mean velocity profile of the approaching flow, was 0.0002m.

### 2.3 Measurement details

**Fig. 3:**
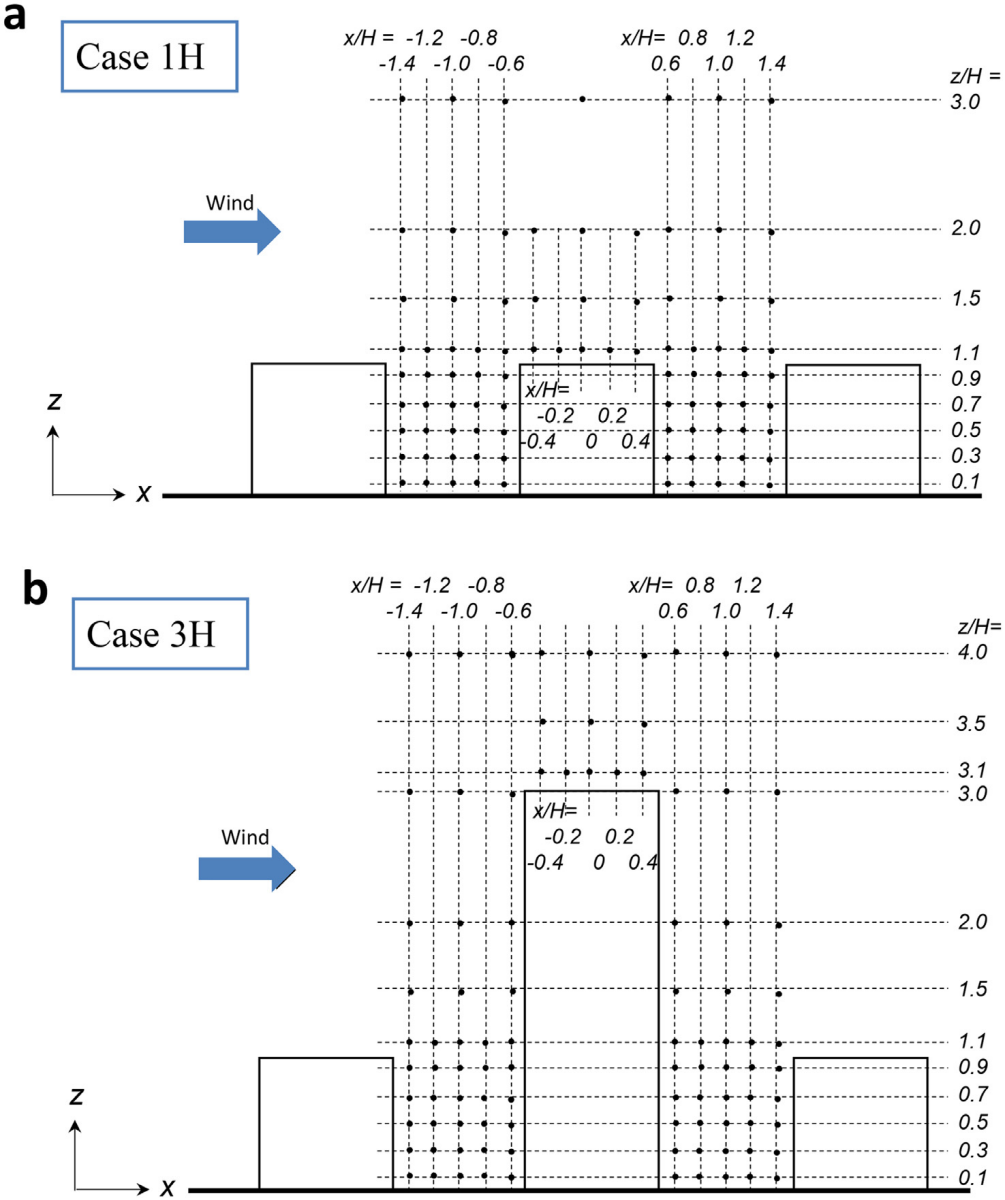

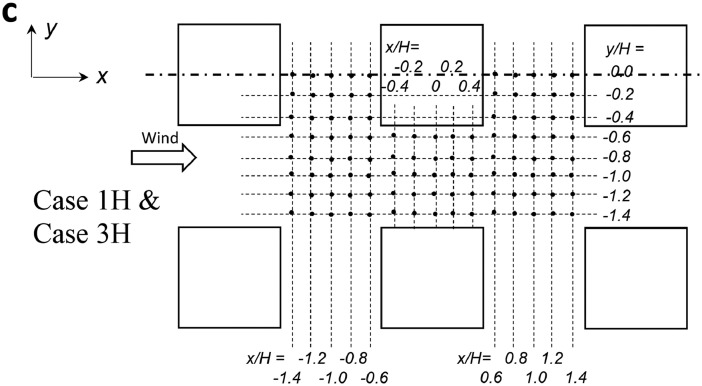
Measurement points (modified from Tominaga and Shirzadi [1]).

*Velocity measurements***:** Three components of the instantaneous velocity vector $u_{i}$ (i stands for x, y, and z, for the streamwise (x), lateral (y), and vertical (z) directions) were measured with a SFP (Dantec Dynamics; 55R55) and a CTA module (Dantec Dynamics; 90C10). The measurement points for the velocity measurement are shown in Fig. 3. The velocity components were measured at 90 and 95 points over the central vertical plane $\left(\frac{y}{H}=0\right)$ in Cases 1H and 3H, respectively. By considering the symmetric flow along the y-axis, 105 measurement points were set over half a horizontal plane $\left(\frac{z}{H}=0.01\right)$ in Cases 1H and 3H.

The three velocity components were measured by rotating the probe in the corresponding direction. Time-averaging was conducted with a sampling rate of 1000 Hz for a period of 60 s to obtain statistically stationary values for the time-averaged velocity components ($U_{i}$) and fluctuating velocity components $u_{i}^{\prime }$, which are calculated as $u_{i}^{\prime }=u_{i}-U_{i}$. The turbulent kinetic energy is defined as $k=0.5(\bar{u{{}_{x}^{\prime }}^{2}}+\bar{u{{}_{y}^{\prime }}^{2}}+\bar{u{{}_{z}^{\prime }}^{2}})$, in which the overbar indicates the time-averaging operator.

For calculation of the wind gust factor (GF) over the pedestrian height (z=0.01m), the instantaneous two-dimensional velocity magnitude $\left(v_{m-xy}\right)$ was measured using a different SPF (Dantec Dynamics; 55R56), which measure only x- and y- velocity in the horizontal plane while the z- (vertical) component is not included, i.e. $v_{m-\mathrm{xy}}=\sqrt{{u_{x}}^{2}+{u_{y}}^{2}}$. GF is calculated at each measurement location as follows:

(2) $$GF=\frac{{\tilde{v_{m-xy\;}}}^{T_{g}}{}_{max}}{{\bar{V_{m-xy\;}}}^{T}}$$

where ${\tilde{v_{m-xy\;}}}^{T_{g}}{}_{max}$ is the maximum moving-averaged velocity magnitude for a gust duration $\left(T_{g}\right)$ of 0.032 seconds in wind tunnel scale (equivalent to 3 seconds in full scale) and ${\bar{V_{m-xy\;}}}^{T}$is the time-averaged velocity magnitude for a sample length (T) of 6.48 seconds in wind tunnel scale (equivalent to 10 minutes in the full scale). The instantaneous two-dimensional velocity magnitude was measured with a sampling rate of 1000Hz for a period of 60 seconds. The time series was divided into 10 segments according to the sample length T to obtain the ${\tilde{v_{m-xy\;}}}^{T_{g}}{}_{max}$ for each segment. The final GF is the average of GF for all segments. The measurement relative expanded uncertainty [15] of the velocity components was less than 10%.

*Wind pressure measurements***:** The static pressures on the building surfaces were measured using a multipoint transducer (Kyowa Electronic Instruments; F94-2206). Long, thin tubes were used to connect the taps to the transducers located outside the wind tunnel. Time-averaging was conducted with a sampling rate of 100Hz for a period of 60 seconds. The pressure coefficient $C_{p}$ is defined as: $C_{p}=\left(P-P_{ref}\right)/\frac{1}{2}\rho U_{H}^{2}$, where P and $P_{ref}$ are the static pressure over the building and the reference static pressure at a point not affected by the building, respectively, and ρ is the air density. The measurement relative expanded uncertainty [15] of the pressure coefficient was less than 5%.

## Ethics Statement

There is no ethical issue in this paper.

## CRediT authorship contribution statement

**Yoshihide Tominaga:** Conceptualization, Methodology, Resources, Funding acquisition. **Mohammadreza Shirzadi:** Methodology, Validation, Formal analysis, Investigation, Writing – original draft.

## Declaration of Competing Interest

The authors declare that they have no known competing financial interests or personal relationships which have or could be perceived to have influenced the work reported in this article.

## Appendix Supplementary Materials

Image, application 1

Image, application 2

Image, application 3

Image, application 4

Image, application 5

Image, application 6

Image, application 7
